# Isolation of *Bacillus cereus* Group from the Fecal Material of Turtles.

**DOI:** 10.17912/micropub.biology.001587

**Published:** 2025-05-26

**Authors:** Jenna L. Storz, Durward L. Bevis, Richard W. McLaughlin

**Affiliations:** 1 Gateway Technical College, Kenosha, Wisconsin, United States

## Abstract

Members of the *Bacillus cereus* group can be animal and human pathogens. They can be found in foods, the environment and in animals. In this study, two *B. cereus* group members were isolated in a previous study from the fecal material of endangered Wood Turtles ( *Glyptemys insculpta* ) and three additional isolates found in the fecal material of painted turtles ( *Chrysemys picta* ) were characterized. The genomes of the five isolates were sequenced and the toxicogenic profiles were determined using the virulence factor database. The most common enterotoxin genes found were *BAS3109* , *hblA* , *hblC* , *hblD* , *inhA* , nheA. *nheB* , and *nheC* .

**Table 1. Bacillus cereus group members present in the fecal material of turtles:** Characteristics of the *Bacillus cereus* group isolates used in the study. ND, not determined; -, not present; +, one copy of the gene; ++, two copies of the gene; +++, three copies of the gene.

**Table:** 

Strain	Barcode 8	Barcode 14	Barcode 15	Barcode 17	Barcode 19
Source	Wood Turtle fecal material	Wood Turtle fecal material	Painted Turtle fecal material	Painted Turtle fecal material	Painted Turtle fecal material
MALDI identification	ND	ND	<i>Bacillus cereus thuringiensis</i> PG IV	<i>Bacillus cereus thuringiensis</i> PG IV	<i>Bacillus cereus thuringiensis</i> PG IV
PubMLST identification	<i>Bacillus weidmannii</i>	<i>Bacillus mycoides</i>	<i>Bacillus thuringiensis</i>	<i>Bacillus thuringiensis</i>	<i>Bacillus toyonensis</i>
Average nucleotide identity (%)	96.3	98.85	96.14	96.19	99.44
Pathogenicity score (# of pathogenic families)	0.698 (32)	0.833 (58)	0.81 (59)	0.815 (80)	0.629 (20)
Restriction modification system	-	-	-	-	-
Hemolysis	Beta	Beta	Gamma	Beta	Beta
Toxigenic profile	Barcode 8	Barcode 14	Barcode 15	Barcode 17	Barcode 19
<i>BAS3109	+	++	+	+	+++
<i>cytK</i>	-	-	+	-	-
<i>hblA</i>	+	+	+	+	+
<i>hblC</i>	+	+	+	+	+
<i>hblD</i>	+	+	+	+	+
<i>inhA</i>	+	+	+	+	+
<i>nheA</i>	+	+	+	+	-
<i>nheB</i>	-	+	+	+	+
<i>nheC</i>	+	+	+	+	+

## Description

Painted turtles ( *Chrysemys picta* ) are very abundant throughout North America (Ernst and Lovich, 2009). Interestingly, this animal which is found in ponds and streams often hibernates for long periods of time in frozen ponds without access to oxygen (Ultsch, 1989). On the other hand, the wood turtle ( *Glyptemys insculpta* ) is considered an endangered species with a declining population (IUCN, 2010).

It has been shown that Chinese soft-shelled turtles' (CSST) ( *Pelodiscus sinensis* ) gut microbiota undergoes significant changes between summer months and hibernation periods (Zhang et al., 2025). It has been suggested that these hibernation changes in the gut microbiota predisposes them to different pathogens including saprophytic pathogens like *Bacillus* spp. (Tao et al., 2024). It has been documented that *Bacillus thuringiensis* , a member of the *Bacillus cereus* group, caused a large-scale fatal infection of CSSTs living in Zhejiang province, China (Chen et al., 2014). In another outbreak involving CSSTs, *B. cereus* strain BC12 was identified as the causative agent (Zhang et al., 2022). Members of the *B. cereus* group are gram-positive, endospore forming bacteria that are environmentally ubiquitous (Liu et al., 2017). This group contains several different species, such as *B. cereus sensu* *stricto* ( *B. cereus* *s. s.* ), *B. anthracis* , *B. albus* , *B. bingmayongensis* , *B. cytotoxicus, B. gaemokensis, B. luti, B. mobilis* , *B. mycoides* , *B. nitratireducens* , *B. pacificus* , *B. paranthracis* , *B. paramycoides* , *B. proteolyticus* , *B. pseudomycoides* , *B. thuringiensis* , *B. toyonensis* , and *B. weihenstephanensis* (Carroll et al., 2020a,b). Some *B. cereus s.s.* strains are able to cause diarrhea that is generally self-limiting. However, other strains are capable of causing more serious illnesses such as anthrax (Mock and Fouet, 2001), meningitis (Gaur et al., 2001), and cutaneous infections (Carroll et al., 2019; Glasset et al., 2016).

According to the World Health Organization (WHO) members of the *B. cereus* group account for approximately 1.4% to 12% of foodborne outbreaks (FBOs) globally (Kirk et al., 2015). “The European Union One Health 2022 Zoonoses Report” recorded numerous cases of FBOs involving bacterial toxins. Toxins produced by members of the *B. cereus* group were among the most prevalent and unfortunately contributed to the deaths of two individuals (Authority EFSA EFS, European Centre for Disease Prevention and Control ECDC, 2023). The main enterotoxins that cause diarrhea are hemolysin BL (Hbl), nonhemolytic enterotoxin (Nhe), and cytotoxin K (CytK). They are pore forming enterotoxins that are made ribosomally (Didouh et al., 2023; Ehling-Schulz et al., 2005). CytK is a toxin that is both hemolytic and necrotic and is capable of producing pores in a phospholipid bilayer (Fagerlund et al., 2004).

Members of the *B. cereus* group are of particular interest in both food safety and public health. They are found throughout the environment (Tirloni et al., 2022) including in raw milk, dairy farms (Meng et al., 2022) and in the intestines of various animals which can result in soil contamination (Tirloni et al., 2022). Since it is well known that reptiles can be a reservoir for pathogens. Perhaps turtles harbors members of the *B. cereus* group that could be potential human pathogens. In a previous study members of the *B. cereus* group were isolated from the fecal material of wood turtles and were evaluated for the presence of enterotoxin genes ( *nheA, entFM* , *hblC* , *cytK* ) using PCR (Nfor et al., 2015). In this present study members of the *B. cereus* group were isolated using the ethanol shock method from the fecal material of painted turtles living in the wild. In total, 32 isolates were screened using MALDI-TOF mass spectrometry (MALDI) and three isolates were found to be members of the *B. cereus* group. MALDI is rapid, but differentiation between members of the *B. cereus* group can be difficult (Pauker et al., 2018; Manzulli et al., 2021). The genomes of members of the *B. cereus* group, as determined by MALDI, were therefore subject to whole genome sequencing (WGS). In addition, the genome of two isolates from a previous study involving wood turtles (Nfor et al., 2015) were also subject to WGS. Identification was determined by PubMLST and then verified by average nucleotide identity (ANI). ANI is a good measure of the genetic distance between bacterial strains (Konstantinidis and Tiedje, 2007) with a purposed species boundary cut-off of 95–96% (Richter and Rosselló-Móra, 2009). The two isolates from the wood turtle were *B. weidmannii* and *B. mycoides.* Two isolates from the painted turtle were identified as *B. thuringiensis* and a third was identified as *B. toyonensis* (Figure 1) *.* No plasmid replicons were found.

Using PathogenFinder-1.1 the isolates in our study were predicted to be human pathogens (Figure 1). Using the virulence finder database the toxigenic profiles were determined (Figure 1). Their profile is similar to the profile of a highly pathogenic *B. cereus* strain (Y271) that was isolated from a diseased CSST living on a farm in Hubei Province, China (Xiao et al., 2023). Strain Y271 contained the Hemolysin BL ( *hblA* , *hblC* , and *hblD* ), Non-hemolytic enterotoxin, NHE ( *nheA* , *nheB* , and *nheC* ), and Enterotoxin FM ( *entFM* ) genes and showed beta hemolysis (Xiao et al., 2023). None of the isolates in this study contained the Enterotoxin FM genes (Figure 1), but some exhibited beta hemolysis (Figure 1). Overall, very few studies have confirmed the presence of members of the *B. cereus* group in reptile species (Nfor et al., 2015; Skóra et al., 2022; Xiao et al., 2023).

Lastly, the isolates were examined for the presence of a restriction modification system (RMS) using Restriction–ModificationFinder-1.1. A RMS is a type of innate immunity, and it is commonly found in prokaryotes. These systems must be able to distinguish between self-DNA and foreign-DNA (Tock et al., 2005). In a recent study, 6354 genomes from members of the *B. cereus* group were examined for the presence of antiviral defense systems. The most common strategy was the use of a RMS (July and Gillis, 2025). In our study none of the genomes contained a RMS (Figure 1).

In the future, fecal samples will be collected and examined for the prevalence of members of the *B. cereus* group from reptiles living in the zoo and in the wild. Animal isolates will be compared to clinical isolates present in the Bacterial and Viral Bioinformatics Resource Center (BV-BRC) database.

## Methods

Animals and sample collection

The bacterial samples from the wood turtles were from the study by (Nfor et al., 2015) and the fecal samples from the painted turtles were from the study by (Fugate et al., 2020). To isolate endospore forming bacteria from the inner portion of the fecal material of Wood Turtles the ethanol shock method was used (Marler et al., 1992). Afterward, cells were plated on TSA (Hardy Diagnostics) agar. The plates were incubated aerobically at 30℃ for two days. Afterwords, cells were plated on TSA agar to obtain a pure culture.

MALDI-TOF MS analysis

A MALDI Biotyper sirius CA System (Bruker Daltonics) was used in order to identify the bacteria. The sample preparation used was recommended by the manufacturer. A more detailed explanation can be found in the study by (Kerin et al., 2023). A Bruker Bacterial Test Standard was used as the positive. The generated spectrum was analyzed using the MALDI Biotyper software (Bruker Daltonics) against the spectra of bacteria included in the Bruker database. The Bruker Bacterial Test Standard was correctly identified. A minimum log score of 2.00 is required for a high-confidence identification and was used as the standard to identify members of the *B. cereus* group.

Genome sequencing

Genomic DNA from two wood turtle isolates and three painted turtle *B. cereus* group isolates were subject to whole genome sequencing (WGS). Genomic DNA was isolated using the Quick-DNA Fungal/Bacterial Miniprep Kit following the manufacturer instructions (Zymo Research). Libraries were constructed using the Rapid Barcoding Kit 24 V14 (SQK-RBK114.24) (Oxford Nanopore Technologies) and sequenced using a PromethION 2 Solo (Oxford Nanopore Technologies). The genomes were assembled using flye version 2.9.1-b1780 (Kolmogorov et al., 2019) and then annotated using the Rapid Annotation using Subsystem Technology tool kit (RASTtk) (Brettin et al., 2015), that can be found at the Bacterial and Viral Bioinformatics Resource Center (BV-BRC) (https://www.bv-brc.org/). This Whole Genome Shotgun project has been deposited at DDBJ/ENA/GenBank under the accession JBMEQE000000000 to JBMEQI000000000. The version described in this paper is version JBMEQE000000000 to JBMEQI000000000.

Bacterial identification

To identify the bacteria, genomes were submitted to the automated computational pipeline of pubMLST (Jolley et al., 2018). Only 100% identification was accepted. Average nucleotide identity (ANI) was also used for identification (Yoon et al., 2017).

Prediction databases

The pathogenicity of the bacteria was predicted using PathogenFinder-1.1 (https://cge.food.dtu.dk/services/PathogenFinder/) and restriction–modification systems were predicted using Restriction–ModificationFinder-1.1 ( https://cge.food.dtu.dk/services/Restriction-ModificationFinder/ ). Plasmid replicons were predicted using PlasmidFinder 2.1 (Carattoli et al., 2014). Putative virulence factors were predicted using the Virulence Factor Database (VFDB) (Liu et al., 2022). Default settings were used for all databases.

Hemolysis assay

All *B. cereus* group isolates were inoculated onto blood agar plates. The plates were incubated aerobically at 30°C for 48 h.
